# T1 and T2 mapping for evaluation of myocardial involvement in patients with ANCA-associated vasculitides

**DOI:** 10.1186/s12968-016-0315-5

**Published:** 2017-01-06

**Authors:** Simon Greulich, Agnes Mayr, Daniel Kitterer, Joerg Latus, Joerg Henes, Hannah Steubing, Philipp Kaesemann, Alexandru Patrascu, Andreas Greiser, Stefan Groeninger, Niko Braun, M. Dominik Alscher, Udo Sechtem, Heiko Mahrholdt

**Affiliations:** 1 Division of Cardiology, Robert-Bosch-Medical Center, Auerbachstrasse 110, 70376 Stuttgart, Germany; 2 Division of Radiology, University Hospital Innsbruck, Innsbruck, Austria; 3 Division of Nephrology, Department of Internal Medicine, Robert-Bosch-Medical Center, Stuttgart, Germany; 4 Center for Interdisciplinary Clinical Immunology, Rheumatology and Auto-inflammatory Diseases, University Hospital Tuebingen, Tuebingen, Germany; 5 Siemens Healthcare GmbH, Erlangen, Germany

**Keywords:** ANCA-associated vasculitides, Cardiac involvement, CMR, LGE, T1 mapping, T2 mapping

## Abstract

**Background:**

Myocardial involvement in AAV patients might be silent, presenting with no or nonspecific symptoms, normal ECG, and preserved left-ventricular ejection fraction (LV-EF). Since up to 50% of deaths in these patients may be due to myocardial involvement, a reliable diagnostic tool is warranted. In contrast to LGE-CMR, which has its strengths in detecting focal inflammatory or fibrotic processes, recent mapping techniques are able to detect even subtle, diffuse inflammatory or fibrotic processes. Our study sought to investigate ANCA (antineutrophil cytoplasmic antibody) associated vasculitides (AAV) patients for myocardial involvement by a cardiovascular magnetic resonance (CMR) protocol, including late gadolinium enhancement (LGE) and mapping sequences.

**Methods:**

Thirty seven AAV patients were prospectively enrolled and underwent CMR imaging. Twenty healthy volunteers served as controls.

**Results:**

Mean LV-EF was 64%; LGE prevalence of the AAV patients was 43%. AAV patients had higher median native T1 (988 vs. 952 ms, *p* < 0.001), lower post-contrast T1 (488 vs. 524 ms, *p* = 0.03), expanded extracellular volume (ECV) (27.5 vs. 24.5%, *p* < 0.001), and higher T2 (53 vs. 49 ms, *p* < 0.001) compared to controls, with most parameters independent of the LGE status. Native T1 and T2 in AAV patients showed the highest prevalence of abnormally increased values beyond the 95% percentile of controls.

**Conclusion:**

AAV patients demonstrated increased T1, ECV, and T2 values, with native T1 and T2 showing the highest prevalence of values beyond the 95% percentile of normal. Since these findings seem to be independent of LGE, mapping techniques may provide complementary information to LGE-CMR in the assessment of myocardial involvement in patients with AAV.

**Electronic supplementary material:**

The online version of this article (doi:10.1186/s12968-016-0315-5) contains supplementary material, which is available to authorized users.

## Background

ANCA (antineutrophil cytoplasmic antibody)-associated vasculitides (AAV) comprise different types of autoimmune diseases, in which autoantibodies react to proteinase 3 (PR3), or myeloperoxidase (MPO) [1]. As systemic disorders, necrotizing granulomatous lesions may occur in any organ, including the myocardium [2]. EGPA (eosinophilic granulomatosis with polyangiitis, formerly known as Churg-Strauss syndrome) and GPA (granulomatosis with polyangiitis, formerly known as Wegener’s granulomatosis) are both subtypes of AAV. The prevalence of myocardial involvement varies, ranging from 16 to 92% in EGPA, and 6 to 86% in GPA patients [3], depending on different diagnostic methods and disease activity. Patients with myocardial involvement may have no or nonspecific symptoms, normal ECG, and preserved left-ventricular ejection fraction (LV-EF) yet they may nevertheless face life-threatening arrhythmias or end-stage heart failure during the course of the disease [4, 5]. EGPA patients frequently show myocardial granulomas and severe tissue alterations on histopathology [6], and up to 50% of patients die of cardiac causes [7]. This is also true in GPA patients [8]. Since myocardial involvement might be reversible if prompt adequate treatment is initiated [9], a reliable tool for the early detection of myocardial involvement is warranted. Cardiovascular magnetic resonance (CMR) offers not only functional assessment but also excellent tissue characterization by the use of late gadolinium enhancement (LGE) [10]. However, LGE is known to perform best in the detection of focal myocardial processes rather than diffuse fibrotic or inflammatory processes [11]. T1 and T2 mapping could already prove their diagnostic value in the detection of both focal and diffuse myocardial alterations in various cardiomyopathies, including cardiac involvement in rheumatic disorders [12, 13] or inflammatory cardiomyopathies [14], and therefore might be appropriate tools to complement LGE-CMR.

Consequently, aim of our study was to evaluate systematically EGPA and GPA patients for myocardial involvement by a dedicated CMR protocol, including LGE, T1 and T2 mapping.

## Methods

### Patient population

Thirty-seven patients were prospectively enrolled between September 2013 and February 2016 if they fulfilled the following criteria: 1) Diagnosis of EGPA or GPA according to the revised Chapel Hill Consensus Conference nomenclature [15]; and 2) no history of CAD, myocardial infarction and/or prior revascularization; and 3) successful CMR.

Sex and age-matched healthy volunteers (*n* = 20) with no history of inflammatory and cardiovascular disease served as control group. All participants provided a blood sample for measurement of hematocrit. All patients gave written informed consent, and the study protocol was approved by the local ethics committee.

### CMR protocol

ECG gated CMR was performed in breath-hold using a 1.5 T Aera (Siemens, Erlangen, Germany) in line with current recommendations [16]. Cine was performed using a steady-state free-precession (SSFP) sequence. LGE images were acquired on average 5–10 min after contrast, using a segmented inversion recovery gradient echo (IR-GRE) sequence [10]. The contrast dose (gadopentetate) was 0.15 mmol/kg.

A modified Look-Locker inversion recovery prototype sequence (MOLLI) was used for T1 mapping and performed in a single midventricular short-axis (SAX) slice at mid-diastole, prior to and 20 min after contrast [17].

Short-axis T2 mapping was performed in the corresponding midventricular SAX before administration of contrast agent using an ECG-triggered T2-prepared single-shot bSSFP prototype sequence with multiple T2 preparation times [18].

More detailed information on CMR protocol is provided in the Additional file 1.

### CMR analysis

Cine and LGE images were evaluated by experienced observers (S.G., H.M.) as described elsewhere [10]. Extent of LGE (expressed as percentage of myocardial mass) was assessed using QMass software (Medis, Leiden, The Netherlands). The distribution of LGE was characterized as epicardial, intramural, transmural, or subendocardial [10].

Color-coded T1, ECV, and T2 maps were generated based on inline-generated, motion-corrected raw images using QMap software 1.0 (Medis, Leiden, the Netherlands) in a single matching midventricular SAX. Motion-corrected T1 maps were examined for quality in three modalities: 1) raw T1 images 2) T1 maps 3) R^2^ maps. Endo- and epicardial contours were manually drawn by two experienced observers (S.G., A.M.), and then divided into 6 segments using the anterior right-ventricular insertion point as reference. Care was taken to avoid partial-volume effects at the endocardial and epicardial borders for T1, ECV and T2 maps. Global T1, ECV, and T2 values were calculated: T1 values were determined by fitting an exponential model to the measured data [19]. The hematocrit allowed with native and post-contrast T1 measurements of the myocardium and blood pool the calculation of extracellular volume (ECV), using a previously described equation [20]. T2 results were obtained by fitting a 2-parameter intensity-weighted exponential model (no offset term) [21].

### Evaluation of disease activity

The Birmingham Vasculitis Activity Score (BVAS) is a validated tool for assessment of disease activity in patients with different forms of vasculitis. The score includes items grouped into 9 organ systems, only features of active vasculitis are counted. BVAS separates symptoms that are new or worse from those that are persistent. Remission is defined as BVAS of 0, whereas active disease means BVAS ≥1 [22].

### Statistical analysis

Absolute numbers and percentages were computed to describe the patient population. Normally distributed continuous variables were expressed as means (with standard deviation) and skewed variables were presented as medians (with quartiles). Comparisons between groups were made using the Mann-Whitney U test or the Fisher’s exact test, as appropriate. *P*-values (two-tailed) of <0.05 were considered significant. All statistical analyses were performed using SPSS, version 22.0 (IBM Corp., Armonk, NY, USA).

## Results

### Patient characteristics

In total, *n* = 57 subjects were included in this study (Table 1): *n* = 37 AAV patients (22 EGPA, 15 GPA), *n* = 20 controls. At inclusion, AAV patients were 55 ± 16 years of age, predominantly female (65%), and did not differ significantly to controls for age and gender (*p* = 0.52 *p* = 0.69, respectively).

**Table 1:** Baseline characteristics

	All patients *n* = 37	EGPA^a^ *n* = 22	GPA^a^ *n* = 15
Age (yrs)	55 ± 16	54 ± 16	55 ± 16
Gender (male)	13 (35%)	5 (23%)	8 (53%)
Cardiovascular risk factors			
Diabetes	7 (19%)	5 (23%)	2 (13%)
Hypertension	13 (35%)	8 (36%)	5 (33%)
Smoking^b^	13 (35%)	7 (32%)	6 (40%)
Hyperlipidemia	6 (16%)	5 (23%)	1 (7%)
Family history of CVD	11 (30%)	8 (36%)	3 (20%)
Obesity (BMI ≥ 30 kg/m^2^)	9 (24%)	5 (23%)	4 (27%)
Symptoms (multiple possible)			
Angina	6 (16%)	4 (18%)	2 (13%)
Dyspnea	16 (43%)	12 (54%)	4 (27%)
Palpitations	4 (11%)	4 (18%)	-
Syncope	1 (3%)	1 (5%)	-
ECG abnormality	11 (30%)	9 (41%)	2 (13%)
Years since diagnosis			
	3 (0–11)	4 (0–11)	2 (0–11)
< 1	12 (32%)	6 (27%)	6 (40%)
1–4	11 (30%)	5 (23%)	6 (40%)
5–9	11 (30%)	10 (46%)	1 (7%)
≥ 10	3 (8%)	1 (5%)	2 (13%)
Disease Activity			
BVAS	5.5 (1–14)	5 (3–8)	11.5 (0–24)
Hematocrit	0.39 (0.36–0.42)	0.4 (0.38–0.42)	0.38 (0.32–0.42)
Medication			
Beta-blockers	9 (24%)	6 (27%)	3 (20%)
ARB	14 (38%)	7 (32%)	7 (47%)
ASA	5 (14%)	5 (23%)	-
CCB	8 (22%)	4 (18%)	4 (27%)
Statins	3 (8%)	2 (9%)	1 (7%)
Diuretics	8 (22%)	7 (32%)	1 (7%)
Steroids	37 (100%)	22 (100%)	15 (100%)
NSAID	1 (3%)	1 (5%)	-
Antibodies	2 (5%)	1 (5%)	1 (7%)
Cyclophosphamide	17 (46%)	4 (18%)	13 (87%)
Azathioprine	4 (11%)	2 (9%)	2 (13%)
Methotrexate	5 (14%)	4 (18%)	1 (7%)

All values are n (%) or mean ± SD or interquartile ranges. ^a^percentages based on number of EGPA/GPA patients, respectively, ^b^current or ever-smokers. *EGPA* eosinophilic granulomatosis with polyangiitis, *GPA* granulomatosis with polyangiitis, *CVD* cardiovascular disease, *BMI* body mass index, *ECG* electrocardiogram, *BVAS* Birmingham vasculitis activity score, *ARB* angiotensin receptor blockers, *ASA* acetylsalicylic acid, *CCB* calcium channel blockers, *NSAID* nonsteroidal anti-inflammatory drug

Non-specific dyspnea was reported in 43%, other symptoms were rare. ECG abnormalities were detected in 30% (*n* = 11). Median time of disease duration was 3 years; all patients were on steroids during the time of CMR.

Dividing AAV patients in LGE-positives and LGE-negatives revealed similar baseline characteristics between both groups, Table 2.

**Table 2:** Baseline characteristics divided by LGE status

	LGE negative *n* = 21	LGE positive *n* = 16	p
Cardiovascular risk factors			
Diabetes	3 (14%)	4 (25%)	0.41
Hypertension	8 (38%)	5 (31%)	0.67
Smoking^a^	8 (38%)	5 (31%)	0.98
Hyperlipidemia	2 (10%)	4 (25%)	0.21
Family history of CVD	6 (29%)	5 (31%)	0.86
Obesity (BMI ≥ 30 kg/m^2^)	6 (29%)	3 (19%)	0.49
Symptoms (multiple possible)			
Angina	2 (10%)	4 (25%)	0.21
Dyspnea	8 (38%)	8 (50%)	0.55
Palpitations	2 (10%)	2 (13%)	0.72
Syncope	-	1 (6%)	0.25
ECG abnormality	5 (24%)	6 (38%)	0.37
Years since diagnosis			
< 1	8 (38%)	4 (25%)	0.57
1–4	5 (24%)	6 (38%)	0.63
5–9	7 (33%)	4 (25%)	0.67
≥ 10	1 (5%)	2 (13%)	0.39
Disease Activity			
BVAS	4 (1–19)	6 (2.5–12.5)	0.91
Hematocrit	0.39 (0.32–0.41)	0.41 (0.38–0.44)	0.10
Medication			
Beta-blockers	5 (24%)	4 (25%)	0.93
ARB	10 (48%)	4 (25%)	0.16
ASA	-	5 (31%)	
CCB	6 (29%)	2 (13%)	0.24
Statins	1 (5%)	2 (13%)	0.37
Diuretics	4 (19%)	4 (25%)	0.66
Steroids	21 (100%)	16 (100%)	0.16
NSAID	1 (5%)	-	
Antibodies	2 (10%)	-	
Cyclophosphamide	11 (52%)	6 (38%)	0.67
Azathioprine	1 (5%)	3 (19%)	0.18
Methotrexate	2 (10%)	3 (19%)	0.42

All values are n (%) or interquartile ranges. *CVD* cardiovascular disease, *BMI* body mass index, *ECG* electrocardiogram, *BVAS* Birmingham vasculitis activity score, *ARB* angiotensin receptor blockers, *ASA* acetylsalicylic acid, *CCB* calcium channel blockers, *NSAID* nonsteroidal anti-inflammatory drug

^a^Current or ever-smokers

### General CMR results

The mean LV-EF was 64% and did not differ from the control group (*p* = 0.84), Table 3. Functional CMR parameters were similar between AAV patients and controls. LGE was present in 16 (43%) of the AAV group, most commonly occurring in a non-ischemic pattern. All controls were LGE-negative. Dividing patients by LGE status revealed that LV-EF was lower, but still preserved, in LGE-positive AAV patients vs. LGE-negative patients (59% vs. 69%, *p* = 0.01), Table 4.

**Table 3:** CMR findings

	Controls (*n* = 20)	Patients (*n* = 37)	p
LV-EF (%)	65 ± 4	64 ± 10	0.84
LV-EDV (ml)	109 ± 27	122 ± 41	0.38
LV-ESV (ml)	38 ± 13	46 ± 31	0.49
LV-SV	71 ± 15	76 ± 18	0.40
LV-EDD	45 ± 5	48 ± 5	0.08
LA (cm^2^)	20 ± 3	20 ± 5	0.84
IVS (mm)	10 ± 2	10 ± 2	0.11
PA (mm)	24 ± 4	25 ± 3	0.37
LV mass (g)	85 ± 20	90 ± 25	0.67
LGE	-	16 (43%)	
epicardial	-	5 (13%)	
intramural	-	7 (19%)	
transmural	-	-	
subendocardial	-	4 (11%)	
% LV mass	-	6.8 ± 5	
Native T1 (ms)	952 (923–960)	988 (965–1017)	**<0.001**
Post-contrast T1 (ms)	524 (500–529)	488 (468–528)	**0.03**
ECV (%)	24.5 (23–25)	27.5 (26–29)	**<0.001**
T2 (ms)	49 (48–51)	53 (51–55)	**<0.001**

All values are n or mean ± SD or interquartile ranges. *CMR* cardiac magnetic resonance, *LV* left-ventricular, *EF* ejection fraction, *EDV* end-diastolic volume, *ESV* end-systolic volume, *SV* stroke volume, *EDD* end-diastolic diameter, *LA* left atrium, *IVS* interventricular septum, *PA* pulmonary artery, *LGE* late gadolinium enhancement, *ECV* extracellular volume

**Table 4:** CMR findings divided by LGE status

	LGE negative (*n* = 21)	LGE positive (*n* = 16)	p
LV-EF (%)	69 ± 7	59 ± 12	**0.01**
LV-EDV (ml)	117 ± 40	128 ± 44	0.37
LV-ESV (ml)	38 ± 21	56 ± 38	0.06
LV-SV	79 ± 22	72 ± 11	0.43
LV-EDD	47 ± 6	49 ± 4	0.09
LA (cm^2^)	21 ± 5	20 ± 4	0.54
IVS (mm)	10 ± 2	11 ± 2	0.56
PA (mm)	25 ± 4	25 ± 2	0.75
LV mass (g)	90 ± 25	90 ± 24	0.93
LGE per patient	-	16 (100%)	
epicardial	-	6 (37%)	
intramural	-	6 (37%)	
transmural	-	-	
subendocardial	-	4 (25%)	
% LV mass	-	6.8 ± 5	
Native T1 (ms)	1002 (970–1025)	982 (957–998)	0.10
Post-contrast T1 (ms)	501 (478–542)	477 (429–497)	**0.04**
ECV (%)	27 (25–28)	28 (26–32)	0.13
T2 (ms)	51 (50–55)	54 (52–56)	0.14

All values are n or mean ± SD or interquartile ranges. *CMR* cardiac magnetic resonance, *LV* left-ventricular, *EF* ejection fraction, *EDV* end-diastolic volume, *ESV* end-systolic volume, *SV* stroke volume, *EDD* end-diastolic diameter, *LA* left atrium, *IVS* interventricular septum, *PA* pulmonary artery, *LGE* late gadolinium enhancement, *ECV* extracellular volume

CMR findings of EPGA and GPA subgroups can be viewed in Tables 5 and 6.

**Table 5:** CMR findings EGPA

	Controls (*n* = 20)	Patients (*n* = 22)	p
LV-EF (%)	65 ± 4	62 ± 12	0.58
LV-EDV (ml)	109 ± 27	124 ± 48	0.38
LV-ESV (ml)	38 ± 13	51 ± 37	0.35
LV-SV	71 ± 15	74 ± 17	0.76
LV-EDD	45 ± 5	48 ± 6	0.06
LA (cm^2^)	20 ± 3	21 ± 6	0.97
IVS (mm)	10 ± 2	10 ± 2	0.23
PA (mm)	24 ± 4	25 ± 3	0.27
LV mass (g)	85 ± 20	89 ± 25	0.83
LGE per patient	-	9 (41%)	
epicardial	-	3 (14%)	
intramural	-	2 (9%)	
transmural	-	-	
subendocardial	-	4 (18%)	
% LV mass	-	7.1 ± 6	
Native T1 (ms)	952 (923–960)	982 (958–1007)	**0.003**
Post-contrast T1 (ms)	524 (500–529)	481 (462–527)	**0.02**
ECV (%)	24.5 (23–25)	28 (25–31)	**<0.001**
T2 (ms)	49 (48–51)	53 (50–55)	**<0.001**

All values are n or mean ± SD or interquartile ranges. *CMR* cardiac magnetic resonance, *LV* left-ventricular, *EF* ejection fraction, *EDV* end-diastolic volume, *ESV* end-systolic volume, *SV* stroke volume, *EDD* end-diastolic diameter, *LA* left atrium, *IVS* interventricular septum, *PA* pulmonary artery, *LGE* late gadolinium enhancement, *ECV* extracellular volume

**Table 6:** CMR findings GPA

	Controls (*n* = 20)	Patients (*n* = 15)	p
LV-EF (%)	65 ± 4	68 ± 7	0.24
LV-EDV (ml)	109 ± 27	118 ± 28	0.57
LV-ESV (ml)	39 ± 13	39 ± 14	0.91
LV-SV	71 ± 15	80 ± 20	0.19
LV-EDD	45 ± 5	47 ± 4	0.36
LA (cm^2^)	20 ± 3	20 ± 3	0.71
IVS (mm)	10 ± 2	11 ± 2	0.11
PA (mm)	24 ± 4	24 ± 4	0.78
LV mass (g)	85 ± 20	92 ± 25	0.57
LGE per patient	-	7 (47%)	
epicardial	-	3 (20%)	
intramural	-	4 (27%)	
transmural	-	-	
subendocardial	-	-	
% LV mass	-	6.3 ± 4	
Native T1 (ms)	952 (923–960)	1002 (972–1023)	**<0.001**
Post-contrast T1 (ms)	524 (500–529)	490 (477–551)	0.16
ECV (%)	24.5 (23–25)	27 (26–28)	**<0.001**
T2 (ms)	49 (48–51)	53 (51–54)	**<0.001**

All values are n or mean ± SD or interquartile ranges. *CMR* cardiac magnetic resonance, *LV* left-ventricular, *EF* ejection fraction, *EDV* end-diastolic volume, *ESV* end-systolic volume, *SV* stroke volume, *EDD* end-diastolic diameter, *LA* left atrium, *IVS* interventricular septum, *PA* pulmonary artery, *LGE* late gadolinium enhancement, *ECV* extracellular volume

### T1 and ECV results

**Fig. 1:**
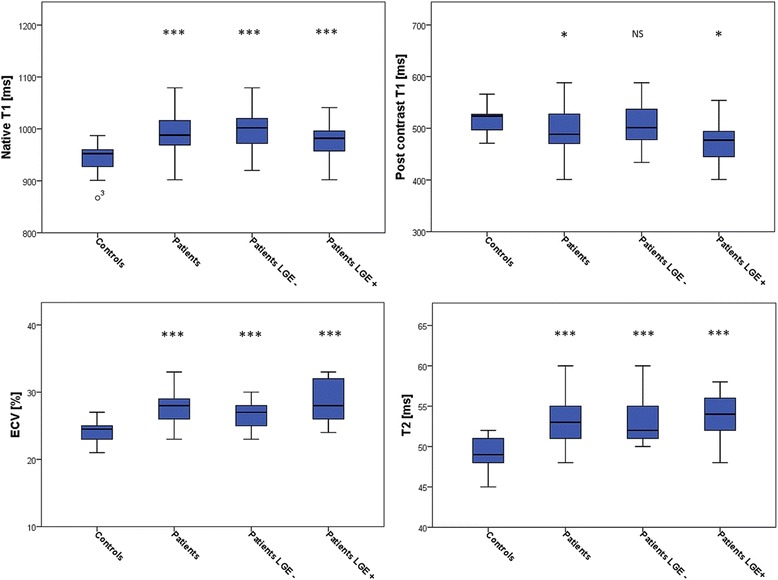
Box plots for median native T1, post-contrast T1, extracellular volume fraction (ECV), and T2 mapping in: controls, AAV patients, late gadolinium enhancement (LGE)-negative AAV patients, and LGE-positive AAV patients. The center line in each box represents the median, whereas the lower and upper limits of each box represent the 25 and 75th percentiles, respectively. Except for post-contrast T1 values between LGE-negative AAV patients and controls, all other parameters (native T1, post-contrast T1, ECV, and T2) differed significantly to the values of controls;*: *p* ≤ 0.05; **: *p* = ≤0.01, ***: *p* ≤ 0.001

We found higher native T1 values in AAV patients than in controls: 988 (965–1017) vs. 952 (923–960) ms, *p* < 0.001; Table 3/Fig. 1. Post-contrast T1 values were decreased in comparison to controls: 488 (468–528) vs. 524 (500–529) ms, *p* = 0.03, Table 3/Fig. 1. For T1-derived extracellular volume fraction (ECV), AAV patients demonstrated significantly higher values: 27.5 (26–29) % vs. 24.5 (23–25) % in the control group, *p* < 0.001, Table 3/ Fig. 1. T1 (native and post-contrast) and ECV values did not correlate with disease activity (BVAS), *p* = 0.38, *p* = 0.09, *p* = 0.21, respectively.

LGE-positive patients showed no significant differences in native T1 and ECV compared to LGE-negative patients (*p* = 0.10, *p* = 0.13, respectively), whereas post-contrast T1 values were significantly lower in LGE-positive patients (*p* = 0.04), Table 4.

Subgroup analysis revealed that, compared to healthy controls, EGPA patients demonstrated: 1) significantly higher median native T1 (*p* = 0.003) and ECV values (*p* < 0.001), 2) significantly decreased post-contrast values (*p* = 0.02), Table 5. Among patients with GPA, we found increased medians for native T1 and ECV values (both *p* < 0.001), but no significant difference for post-contrast T1, *p* = 0.16, Table 6.

Comparing EGPA vs. GPA patients revealed no significant differences in native T1, post-contrast T1 and ECV (*p* = 0.09, *p* = 0.32, *p* = 0.44, respectively).

### T2 results

Higher median myocardial T2 values were reported in AAV patients compared to controls: 53 (51–55) vs. 49 (48–51) ms, *p* < 0.001, Table 3/Fig. 1. LGE-negative patients showed a median of 51 (50–55) ms, and LGE-positive patients of 54 (52–56) ms, *p* < 0.001 each, also see Table 4. Similar to T1, T2 values did not correspond to the patients disease activity (BVAS), *p* = 0.89.

In the subgroup analysis, T2 values for both EGPA and GPA patients differed significantly from the T2 values of the control group (*p* < 0.001 each), Tables 5 and 6. In contrast, T2 values between EGPA and GPA showed no significant difference (*p* = 0.85).

**Fig. 2:**
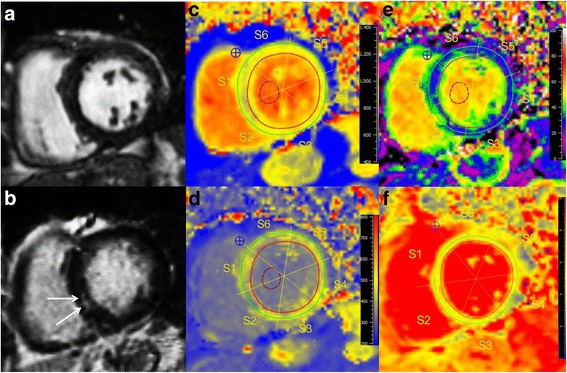
CMR of a 77-year-old female with EGPA (BVAS = 4), presenting with palpitations and atrial fibrillation. Cine images (**a**) revealed normal LV-EF (66%), LGE (**b**) detected intramural enhancement in the inferior septum (*white arrows*), suggestive of cardiac involvement. Native T1 map (**c**) showed increased T1 with 980 ms, shortened post-contrast T1 (**d**) with 452 ms, increased ECV (**e**) of 37%, and higher T2 (**f**) (52 ms) compared to controls

**Fig. 3:**
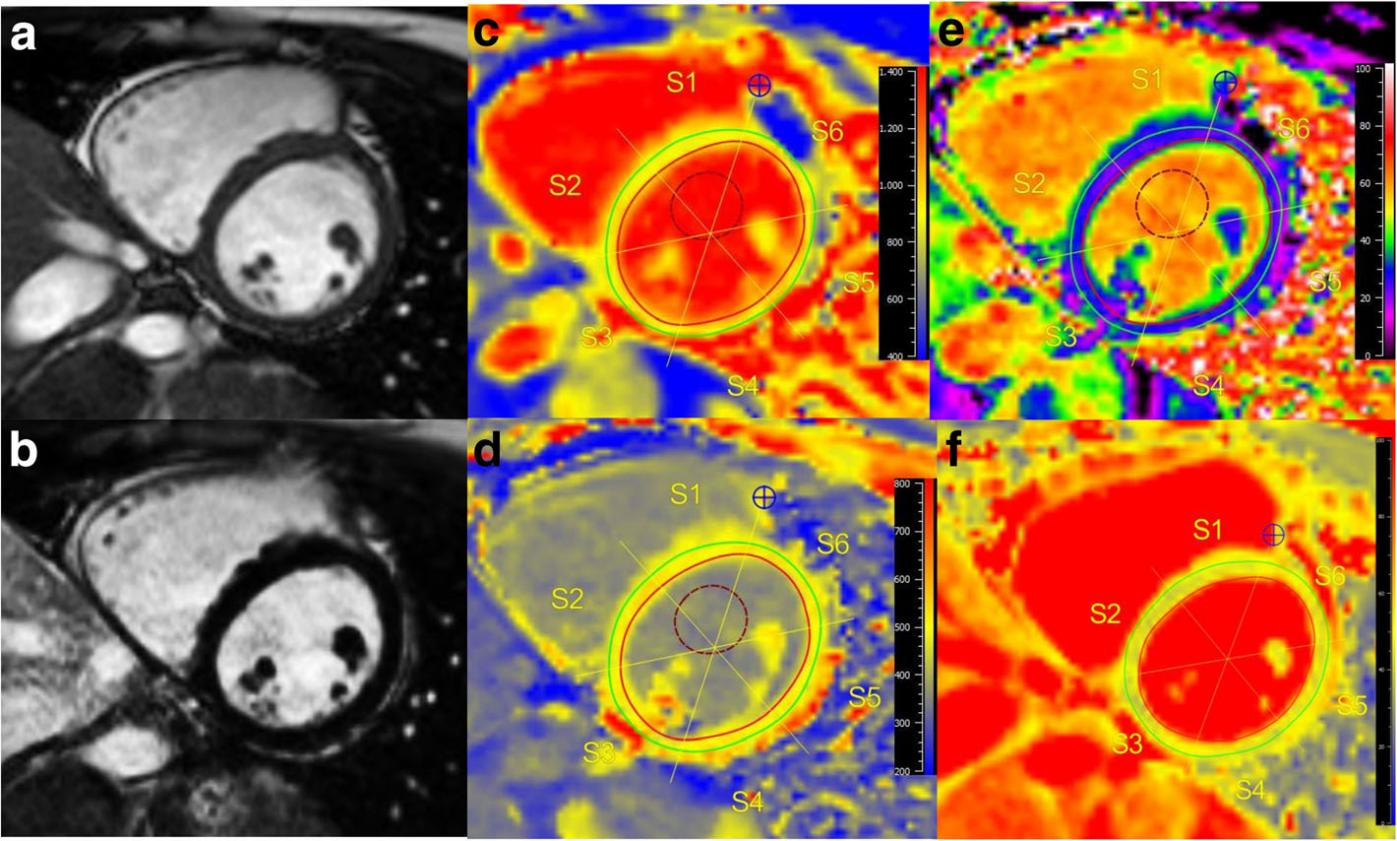
CMR of a 26-year-old female with a history of EGPA for 3 years with the same BVAS (=4) as the patient from Fig. 2. She was suffering from palpitations and dyspnea, ECG was normal. Cine images (**a**) showed a preserved LV-EF (67%), LGE images (**b**) were negative. However, native T1 (1019 ms, **c**), ECV (27%, **e**), and T2 (52 ms, **f**) were increased compared to controls, suggesting myocardial involvement despite normal LV-EF, and unremarkable ECG

Figure 2 displays a female EGPA LGE-positive patient with increased native T1, ECV, and T2, and decreased post-contrast T1. Figure 3 illustrates a LGE-negative EGPA patient with increased T1, ECV, and T2 values.

### Values above the 95% percentile of normal

**Fig. 4:**
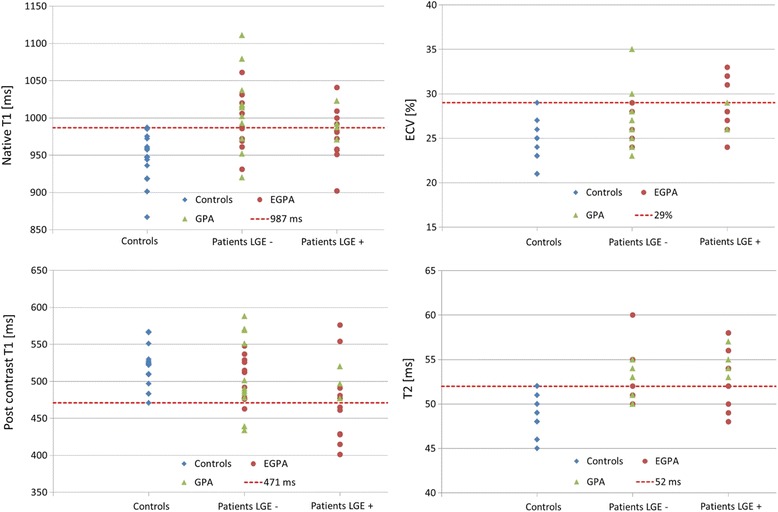
Values for T1 (native, post-contrast), ECV, and T2 in controls, LGE-negative and LGE-positive EGPA (represented by *circles*), and GPA (represented by *triangles*) patients. The dotted lines represent the thresholds for values beyond the 95% percentile of the control group (definite abnormal values)

Definite abnormal values (beyond the 95% percentile of controls) were above 987 ms for native T1, below 471 ms for post-contrast T1, above 29% for ECV, and above 52 ms for T2, Fig. 4.

Fifty-one percent (*n* = 19) of the AAV patients showed a native T1 value above the 95% percentile of controls, with the majority (68%) of them being LGE-negative. In *n* = 10 patients, post-contrast values were below 471 ms, 30% of these were LGE-negative. Measurement of ECV revealed that *n* = 7 patients had definite abnormal values, 29% LGE-negative. 54% (*n* = 20) of the AAV patients showed definite abnormal T2 values, 45% LGE-negative.

Patients with EGPA showed in 40% (9 out of 22 EGPA patients) an increased native T1 value above the 95% percentile of controls, 56% LGE-negative. Eight patients (1 LGE-negative) had post-contrast T1 values lower, and five patients (all LGE-positive) had ECV values higher than the 95% percentile of controls. 50% (*n* = 11) of the EGPA patients showed definite abnormal T2 values, 36% were LGE negative.

Patients with GPA showed in 67% (10 out of 15 GPA patients) increased native T1 values above the 95% percentile of controls, with 80% classified as LGE-negative. Two patients (both LGE-negative) had post-contrast T1 values lower, and two patients (both LGE-negative) had ECV values higher than the 95% percentile of controls. 60% (9 out of 15 GPA patients) showed definite abnormal T2 values: 56% were LGE negative.

### AAV patients with ECG abnormality

In AAV patients with ECG abnormalities (*n* = 11) functional CMR parameters, LGE prevalence, native and post-contrast T1 did not differ significantly to patients with no ECG abnormalities, Table 7. However, ECV and T2 mapping revealed higher median values in patients with ECG abnormality (ECV 29%, T2 55 ms) vs. patients with unremarkable ECG (ECV 26%, T2 52 ms), *p* = 0.01 and *p* = 0.009, respectively. 36% of the patients with ECG abnormalities, and 11% of the patients with normal ECG showed ECV values above the 95% percentile of controls. Furthermore, 82% of the patients with ECG abnormalities (9 out of 11) demonstrated increased T2 value above the 95% percentile of normal compared to 42% (11 out of 26) of the patients with unremarkable ECG.

**Table 7:** CMR findings in patients with normal ECG vs. abnormal ECG

	ECG normal (*n* = 26)	ECG abnormal (*n* = 11)	p
LV-EF (%)	66 ± 9	61 ± 12	0.47
LV-EDV (ml)	116 ± 28	136 ± 60	0.68
LV-ESV (ml)	40 ± 17	59 ± 47	0.46
LV-SV	76 ± 17	77 ± 21	0.79
LV-EDD	47 ± 5	49 ± 6	0.31
LA (cm^2^)	19 ± 3	23 ± 7	0.16
IVS (mm)	10 ± 2	11 ± 2	0.06
PA (mm)	25 ± 3	25 ± 4	0.80
LV mass (g)	91 ± 23	89 ± 30	0.46
LGE positive (n)	10 (39%)	6 (55%)	0.37
Native T1 (ms)	983 (960–1010)	1006 (983–1037)	0.11
Post-contrast T1 (ms)	487 (464–538)	490 (476–512)	0.62
ECV (%)	26 (25–28)	29 (28–32)	**0.01**
T2 (ms)	52 (50–54)	55 (53–57)	**0.009**

All values are n or mean ± SD or interquartile ranges. *CMR* cardiac magnetic resonance, *LV* left-ventricular, *EF* ejection fraction, *EDV* end-diastolic volume, *ESV* end-systolic volume, *SV* stroke volume, *EDD* end-diastolic diameter, *LA* left atrium, *IVS* interventricular septum, *PA* pulmonary artery, *LGE* late gadolinium enhancement, *ECV* extracellular volume

## Discussion

This is the first study evaluating cardiac involvement in patients with AAV by a CMR protocol, including LGE, and T1 and T2 mapping techniques. Major findings are: 1) Patients with AAV show increased native T1, ECV, T2 and decreased post-contrast T1 values compared to controls. 2) These findings are independent of the presence of LGE for native T1, ECV, and T2. 3) Most significant differences to healthy controls were detected for native T1, ECV, and T2. 4) Native T1 and T2 mapping were the most frequent parameters above the 95% percentile of controls. 5) AAV patients with ECG abnormality show higher ECV and T2 values compared to patients with normal ECG.

### Patient characteristics

The majority of patients were middle-aged, and non- to oligosymptomatic [3, 23]. ECG abnormalities were detected in 30%, and were more common in EGPA patients than in GPA patients, which concur the results from another study investigating cardiac involvement in AAV patients [3].

### General CMR results

The mean LV-EF was preserved (64%), and cardiac dimensions were similar to the healthy control group, underlining the necessity of further detailed tissue characterization. LGE, highly suggestive of myocardial involvement, was present in almost half of the AAV patients (43%) in a mainly non-ischemic pattern (epicardial and/or intramural). However, 18% of the EGPA patients demonstrated the specific subendocardial LGE pattern, not related to a distinct coronary territory [4, 9]. Hazebroek [3] investigated 50 EGPA and 41 GPA patients for cardiac involvement and found a prevalence of LGE in 22% of the EGPA, and 19% of the GPA patients. In contrast, we observed a higher LGE prevalence of 45% in EGPA, which is in line with histologic findings [6], and of 25% in the GPA patients. A reason for this discrepancy might be that the latter study only included patients in sustained remission (BVAS = 0), whereas our AAV patients had active disease (BVAS = 5.5).

### T1 and ECV results

Native T1 and ECV are supposed to represent myocardial fibrosis in the absence of edema, infiltration or infarction, which could be demonstrated by various studies, including inflammatory cardiomyopathies [14], and rheumatic disorders, such as systemic lupus erythematosus, and rheumatoid arthritis [12, 13]. Since post-contrast T1 depends on renal function, body fat distribution, dose of contrast agent, and the time delay between application of contrast agent and measurement of values, there is consensus that ECV may overcome these limitations by considering the pre- and post-contrast myocardial and blood T1, and adjusting for the hematocrit [11, 17]. We observed higher native T1 and ECV values in AAV patients than in controls, both *p* < 0.001, Table 3/Fig. 1, supporting data from other studies in patients with rheumatic disorders [12, 13]. Ntusi et al. [13] demonstrated in patients with rheumatoid arthritis higher native T1 and ECV compared to controls, both *p* < 0.001, matching our results. Furthermore, post-contrast T1 values were decreased in comparison to controls (*p* = 0.04), which is another similarity to our AAV population, *p* = 0.03. For native T1 and ECV, values of LGE-positive AAV patients showed no significant differences to the values of LGE-negative ANCA patients (*p* = 0.10, *p* = 0.12, respectively), completely in line with the latter studies [12, 13].

In the EGPA subgroup, patients vs. controls demonstrated: 1) significantly higher median native T1 (*p* = 0.003) and ECV values (*p* < 0.001), 2) significantly decreased post-contrast values (*p* = 0.02). In EGPA, release of toxic mediators by activated eosinophils can cause myocardial alterations, and the presence of myocardial granulomas might yield scar tissue [23], resulting in a combination of both inflammation (active phase) and fibrosis (more chronic phase) [4].

For GPA, we also found increased medians for native T1 and ECV values in comparison to controls (both *p* < 0.001). Similar to EGPA, a combination of inflammation and fibrosis is suggested for patients with GPA [24], which is further underlined by the finding that values of both AAV subgroups (EGPA vs. GPA) revealed no significant differences in native T1, post-contrast T1, and ECV values (*p* = 0.09, *p* = 0.32, *p* = 0.44, respectively). Moreover, Hazebroek et al. [3] detected inflammatory states in most of the endomyocardial biopsy specimens of EGPA and GPA patients, and found no significant difference in interstitial fibrosis quantified as collagen volume fraction between EGPA and GPA (*p* = 0.91), supporting our results.

### T2 results

T2 allows detection of the myocardial free tissue water content [14], predisposing this technique for detection of active myocardial inflammation. Inflammation is one of the hallmarks in patients with systemic vasculitis, indicating a more acute phase of the disease, which might resolve after appropriate treatment. In contrast, fibrotic lesions detected by LGE-CMR tend to represent a more chronic phase, persisting on follow-up exams [25, 26]. Therefore, T2 mapping might help to separate myocardial inflammation from myocardial fibrosis since T2 mapping has proven to be an objective, precise and reliable method for the detection of inflammatory conditions [18, 27].

As to expect, we found higher median myocardial T2 values in AAV patients compared to controls, *p* < 0.001, Table 3/Fig. 1. Similar to native T1 and ECV, this finding was independent of the patients’ LGE status, suggesting an additional gain of information about the patients’ inflammatory status compared to the assessment with LGE-CMR alone.

In the subgroup analysis, T2 values for both EGPA and GPA patients differed significantly from the T2 values of the control group (*p* < 0.001 each), Tables 5 and 6. In contrast, T2 values between both AAV subgroups (EGPA and GPA) showed no significant difference (*p* = 0.85). These results suggest a common pathway of myocardial involvement in AAV patients, reflecting a combination of both ongoing inflammation and fibrosis, which is supported by histology in the literature [3, 25].

### Values above the 95% percentile of normal

Despite highly significant differences between patients and controls, there is some overlap in T1, ECV, and T2 values, which seem to lower the diagnostic accuracy in the individual AAV patient. Defining the 95% percentile of the control group as threshold for definite abnormal values, we found native T1 and T2 to be the most frequent values: 51% (*n* = 19) of the AAV patients showed a native T1 value above the 95% percentile of the control group, with the majority (68%) of them reported LGE-negative, suggesting potential diagnostic value in patients with no overt cardiac involvement. 54% (*n* = 20) of the AAV patients showed definite abnormal T2 values, with 45% LGE-negatives, underscoring that increased T2 values are not driven by the presence of LGE alone.

Although the patients’ disease activity (BVAS) did not correlate to the mapping values in our study, GPA patients demonstrated compared to EGPA patients: 1) higher BVAS (11.5 vs. 5), and 2) higher percentage of patients with abnormal values beyond the 95% percentile of normal, potentially reflecting higher disease activity. However, it must be noted that myocardial involvement may be the initial manifestation of AAV, which might be subclinical in early stages.

### AAV patients with ECG abnormality

AAV patients with ECG abnormalities showed significantly increased ECV values, but not a significantly higher prevalence of LGE compared to patients with normal ECG, *p* = 0.01, *p* = 0.37, respectively. Therefore, increased ECV values may reflect diffuse fibrosis (in addition to focal fibrosis detected by LGE-CMR), potentially yielding ECG abnormalities. In a recent study [28], histological ECV of left-ventricular specimens was significantly correlated with CMR-ECV (*r* = 0.493, *p* = 0.002). Of note, patients with higher ECV demonstrated a higher prevalence of ECG abnormalities (*p* < 0.001), supporting our findings.

Additionally, patients with ECG abnormalities showed higher T2 values than patients without ECG abnormalities (*p* = 0.009), which is a similarity to a study performed in patients with sarcoidosis [29]. Of note, 82% of the patients with ECG abnormalities in our study demonstrated increased T2 value above the 95% percentile of normal. Since increased T2 values, as a substrate of inflammation, are supposed to represent potentially reversible processes [29], T2 mapping might serve as an appropriate quantitative biomarker during the clinical course of inflammatory disease such as AAV.

### Clinical implications

Our data demonstrate that patients with AAV (EGPA and GPA) show several abnormalities detected by CMR mapping techniques. Interestingly, most patients were non- or oligosymptomatic, had normal ECG, and a preserved LV-EF, suggesting an incremental value of mapping techniques in the assessment of subclinical myocardial involvement in early, potentially reversible stages of AAV, which might have been missed otherwise. Since native T1, ECV, and T2 values were independent from the presence of LGE, these parameters seem to provide complementary information about diffuse myocardial involvement compared to LGE alone. Most significant differences (beyond the 95% percentile of controls) were observed for native T1 and T2, suggesting a combination of both chronic (fibrosis) and acute (inflammation) stages in AAV patients.

Our findings suggest that recent T1 and T2 mapping techniques might play a role in patients with AAV for: 1) detection of even subtle myocardial involvement, 2) assessment of different stages of the disease (acute vs. chronic), 3) decision-making about subsequent medical therapy, preventing progression of further cardiac damage, and 4) the evaluation of response to treatment during follow-up.

### Limitations

Since this is a single-center study, potential center-specific bias cannot be excluded. The measurement of global myocardial T1 or T2 values in a single mid-ventricular slice might miss a focal process due to “averaged” values. However, this approach is common practice [30], less subjective than individualized regions of interest, and might be better comparable to potential follow-up exams.

Endomyocardial biopsy (EMB) was not routinely performed. However, it is known that EMB has several limitations, lowering its diagnostic benefit. Furthermore, in non- to oligosymptomatic patients with preserved LV-EF and negative LGE-CMR, this would be a rather unethical approach.

Whether abnormalities by T1 and T2 mapping in patients with AAV represent myocardial involvement should be evaluated by further studies, including EMB. However, T1 and T2 mapping seem to be appropriate techniques, since a combination of both inflammation and fibrosis could be detected by histology in other studies with AAV patients [3, 25].

## Conclusions

In patients with AAV (EGPA and GPA), we found higher values for native T1, ECV, and T2 compared to controls, irrespective of the presence of LGE. Native T1 and T2, assessing diffuse fibrotic and inflammatory myocardial changes, were the most frequent parameters showing values beyond the 95% percentile of controls. Therefore, they seem to be the most promising techniques for the early detection of myocardial involvement in potential reversible stages.

However, further studies are needed, elucidating the role of mapping techniques in diagnosis and monitoring of myocardial involvement in patients with AAV.

## Additional file

Additional file 1: “Characteristics of the T1 mapping and T2 mapping sequences”. (DOC 25 kb)

## Abbreviations

AAV: ANCA-associated vasculitides
ANCA: Anti-neutrophil cytoplasmic antibody
BVAS: Birmingham vasculitis activity score
CAD: Coronary artery disease
CMR: Cardiovascular magnetic resonance
CVD: Cardiovascular disease
ECG: Electrocardiogram
ECV: Extracellular volume
EF: Ejection fraction
EGPA: Eosinophilic granulomatosis with polyangiitis
GPA: Granulomatosis with polyangiitis
IQR: Interquartile range
IVS: Interventricular septum
LA: Left atrium
LGE: Late gadolinium enhancement
LV: Left ventricle
LV-EDV: Left-ventricular end-diastolic volume
LV-EF: Left-ventricular ejection fraction
LV-ESV: Left-ventricular end-systolic volume
MOLLI: Modified look-locker inversion recovery sequence
MPO: Myeloperoxidase
NYHA: New York Heart Association
PR3: Proteinase 3
SAX: Short axis
SSFP: Steady-state free-precession
